# Origin of MMP-8 and Lactoferrin levels from gingival crevicular fluid, salivary glands and whole saliva

**DOI:** 10.1186/s12903-021-01743-5

**Published:** 2021-08-05

**Authors:** Liza L. Ramenzoni, Deborah Hofer, Alex Solderer, Daniel Wiedemeier, Thomas Attin, Patrick R. Schmidlin

**Affiliations:** 1 Clinic of Conservative and Preventive Dentistry, Center of Dental Medicine, University of Zurich, Plattenstrasse 11, 8032 Zurich, Switzerland; 2 Laboratory of Applied Periodontal and Peri-Implantitis Sciences, Clinic of Conservative and Preventive Dentistry, Center of Dental Medicine, University of Zurich, Plattenstrasse 11, 8032 Zurich, Switzerland; 3 Statistical Services, Center of Dental Medicine, University of Zurich, Plattenstrasse 11, 8032 Zurich, Switzerland

**Keywords:** Matrix metalloproteinase-8, Lactoferrin, Biological markers, Periodontal pathogens, Gingival crevicular fluid, Chronic periodontitis

## Abstract

**Background:**

Pathologically elevated levels of matrix metalloproteinase-8 (MMP-8) and Lactoferrin in oral fluids have been associated with the presence of gingivitis/periodontitis. This study aimed to assess the origin of MMP-8 and Lactoferrin in periodontitis patients and to identify the degree to which conventional clinical parameters correlate with their presence.

**Methods:**

A total of ten periodontitis and ten healthy patients were included in this study. Whole saliva (stimulated and unstimulated), parotid/sublingual glandular fluid and gingival crevicular fluid from pockets and sulci were tested for MMP-8 and Lactoferrin and protein concentrations were quantified using an ELISA assay. Clinical parameters were checked for potential associations with MMP-8 and Lactoferrin levels.

**Results:**

Periodontal patients presented higher concentrations of MMP-8 and Lactoferrin in pockets than other sources (*P* = 0.03). Lactoferrin measurement was higher in the parotid compared to sublingual glandular fluid in periodontitis patients (*P* = 0.03). Increased probing pocket depth was positively correlated with high MMP-8 and Lactoferrin levels.

**Conclusions:**

Periodontal pockets appear to be the major source of active matrix metalloproteinase and Lactoferrin, which also may also enter the oral cavity through the salivary glands. Since clinically healthy sites in periodontitis patients also had elevated biomarker levels, gingival crevicular fluid biomarker testing may be more predictive of future tissue breakdown than conventional clinical parameters.

## Background

Periodontitis is a common inflammatory oral condition induced by certain periodontal bacterial species [1]. These bacteria colonize the non-shedding hard tissue surfaces and when allowed to grow and mature develop into biofilms which may trigger an inflammation of the periodontal tissues i.e. gingiva, periodontal ligament and alveolar bone [1, 2]. The severity ranges from superficial inflammation of the gingiva (gingivitis) to extensive destruction of the connective tissue and bone (periodontitis), finally leading to tooth loss if untreated [2]. The prevalence of periodontitis is high, with an average impact on 46% of the adult population in developed countries and higher incidence in developing nations [3]. Peri-implantitis is also considered a periodontitis-like disease process, where similar periodontitis bacterial plaque is regarded as its primary etiologic factor in the loss implants. In fact, the literature indicates that the presence or history of periodontitis may be one of the highest risk factors for peri-implantitis [4]. Clinical findings around tooth with periodontitis and failing implants include marked gingival inflammation, deep pocket formation, and progressive bone loss [4]. As both periodontitis and peri-implantitis have similar inflammatory phenotypes when assessed cross-sectionally, treatment protocols for peri-implantitis were modeled according to those used for periodontitis. Often, periodontal disease is not reversable but manageable and scaling root planing treatment may be enough to control periodontal infection, restore oral tissues to good health, and tighten loose teeth. The treatment procedures for peri-implantitis are also very similar to that of periodontitis, but more intense and often surgery based. Treatment for both periodontitis and peri-implantitis, even when successful, will not result in a complete regeneration of lost structure. Therefore, it is crucial to recognize early (subclinical) disease activity before its establishment and assess risk for further disease progression with further tissue destruction.

At a closer look, the destruction of host tissues in periodontitis and peri-implantitis is caused by an interaction between microbial and host factors, which synergistically allow otherwise protective enzymes, proteins and bacteria to modify in ways that become pathogenic and beyond the body’s innate ability to hold inflammatory destruction at bay [5]. The term “proteome” was coined in 1996 to describe the study of changes occurring in the oral environment as microorganisms adapt to environmental changes [6]. Since historic periodontal parameters of pocket depth, recession and bleeding are signs of past destruction, they are of limited value in identifying current (subacute) disease challenges and providing tailored preventive therapy to avoid loss of periodontal tissue [7–10]. Recognizing these facts, many proteomic point-of-care diagnostics biomarkers are being sought to proactively determine the presence or absence of periodontal destruction factors [11]. One well-researched inflammatory biomarker is matrix metalloproteinases 8 (MMP-8), also known as collagenase-2 or neutrophil collagenase [12–15]. It is a predominant collagenase mostly identified in gingival crevicular fluid (GCF) and associated with periodontitis severity, especially in activated/active form (aMMP-8). The major cellular source of MMP-8 are polymorphonuclear neutrophils (PMN) and increased production levels of aMMP-8 signify the progression of gingivitis into periodontitis, with associated soft tissue destruction [16]. In fact, some authors considered MMP-8 as the main collagenase in periodontitis, since 90% to 95% of collagenolytic activity in gingival crevicular fluid and saliva is actually derived from higher levels of MMP-8 compared to healthy individuals [12–16]. Consequently, MMP-8 is regarded by many studies as one of the most promising biomarkers for periodontitis in oral fluids [12–16]. Another key periodontal biomarker is Lactoferrin, which is globular glycoprotein secreted in response to a bacterial challenge, and has also been shown to be strongly associated, especially with periodontitis [16, 17]. In fact, some authors identify increased levels of Lactoferrin in both unstimulated and stimulated saliva in advanced periodontitis patients compared to healthy patients [16–18]. Lactoferrin is known to for its ability to bind iron, which removes important elements involved in oral bacterial cell growth in saliva [16–18]. Thus, Lactoferrin is claimed to have antibacterial activity and low concentrations of Lactoferrin promote bacterial growth. Periodontal biomarkers, such as MMP-8 and Lactoferrin, that are easy to access and can be rapidly and non-invasively sampled, would be of a predictive nature and a great benefit to patients at risk for early developing periodontal disease [19].

Saliva and GCF were previously both cited as sources of aMMP-8 and Lactoferrin, and fit periodontal collection parameters [18–20]. However, saliva is known to have multiple contributing sources (parotid, sublingual and submandibular glandular fluids, as well as GCF washout) [21]. Following the studies on the origin and variation in number of polymorphonuclear/leukocytes in the human saliva, the gingival crevices are suggested to be the main point of entry for PMN and its products into the GCF and oral cavity, however another proportion is originated from salivary glands [22, 23]. The detection of MMP-8 in GCF and whole saliva is the most frequently studied [21–23]. However, the contributions of salivary glands to the MMP-8 enzyme production has not yet been demonstrated. In fact, whole saliva per se represents a complex fluid mixture, and gingival crevice exudate accounts only for a part of its composition, while another important part comes from major salivary glands [24]. Lactoferrin was also shown to be synthesized by exocrine glands and neutrophils in infection/inflamed sites [25, 26]. Nevertheless, the data from whole saliva samples has failed to reveal the other multiple possible origins of MMP-8 and Lactoferrin inflammatory biomarkers in the oral cavity.

Therefore, the aim of the study was not only to determine the presence of aMMP-8 and Lactoferrin in patients newly diagnosed with active chronic periodontal disease, but also to determine the different possible sources of these biomarkers (GCF, major salivary glands and/or whole saliva, both stimulated and unstimulated). We hypothesized that both aMMP-8 and Lactoferrin may be originated from different salivary glands, other than only oral rinse or GCF, since saliva has multiple other contributing sources. In addition, the associations between aMMP-8 and Lactoferrin to clinical parameters of periodontitis (i.e. the clinical extent of periodontitis) and key deep pocket bacteria were also investigated.

## Methods

### Participants and study design

A total of ten periodontitis patients (6 men and 4 women) and ten healthy patients (2 men and 8 women) were recruited for this study. New patients referred to the University of Zurich, Center of Dental Medicine, Clinic of Conservative and Preventive Dentistry for treatment of chronic periodontal disease were asked if they would volunteer to participate in the study, after having been evaluated for their overall treatment needs. The healthy patients were similarly recruited. All volunteers were informed of the aims and parameters of the study, but offered no compensation for participation. Patients were provided with informed written consent and notified of their right to rescind agreement at any time. The study protocol was approved by the Canton of Zurich Ethics Committee (BASEC-Nr. 2016-00243), according to the Helsinki Declaration. The inclusion criteria were as follows: patients between the ages of 18 and 75, with generalized chronic periodontitis, who had at least one tooth site per quadrant with periodontal probing pocket depths (PPD) ≥ 5 mm, bleeding on probing (BOP), plaque index (PI) ≥ 20%, clinical attachment Level (CAL) ≥ 3 mm, furcation involvement, and radiographical bone assessment. All patients had at least 22 teeth and an untreated chronic generalized periodontitis, according to the Periodontology Classification of Periodontal Diseases and Conditions [1], with more than 30% of tooth sites affected (clinically and radiographically). Inclusion criteria for the healthy patients, beside similar age, were no history on periodontal disease treatment, probing pocket depths (PPD) ≤ 3 mm, mean BOP percentages ≤ 25% and no radiographically identified bone loss or CAL. Patients of both genders were eligible to participate. The exclusion criteria for both test groups were as follows: pregnant or nursing women, heavy smokers (> 10 cigarettes/day), Wharton’s duct or Stensen’s duct redness, gingival hypertrophy, antibiotic or anti-inflammatory therapy within the last six months, a history of any systemic disease (i.e. immunosuppressed or diabetes patients) affecting the outcome of the periodontal therapy and/or any periodontal treatment within the previous six months. Two patients (1 man, 1 woman) from the initial periodontal group had to be excluded after antibiotic therapy for a sinus infection and bladder infection within the previous 6 months were belatedly reported. In total, ten periodontitis patients and ten healthy patients participated in the study.

### Clinical evaluation

All parameters, anamneses (medical and dental history) and dental status were obtained by one calibrated examiner (D.H.) between September 2018 and September 2019. This evaluation included: decayed, missing and filled teeth; tilt or overeruption; mobility and sensitivity [21, 27, 28]. A thorough periodontal examination was performed [29] including: assessment of tooth PPD, BOP, PI and relative CAL at each site using a manual probe (PCP10-SE, Hu-Friedy, Chicago, IL, USA), BOP at six sites per tooth, the presence or absence of pus secretion, the presence or absence of gingival recession, the presence or absence of furcation, and the presence or absence of plaque. Periodontitis was diagnosed according to Armitage’s classification whereby clinical attachment loss ≥ 3 mm affecting more than 30% of the dentition was considered generalized moderate to severe periodontitis [1]. Under the new classification system, these patients would be classified as having Stadium III, Grade B periodontitis [30]. Those patients meeting the inclusion criteria were asked if they would be interested in participating in this study. The study was explained to be noninvasive, that their data would be anonymized and that knowledge gained would help with our understanding of the sources of periodontal inflammation and find simplified measures for determining its presence before clinical destruction becomes obvious. Written information about the study was provided and patients were asked not to eat, smoke, drink or rinse his/her mouth for 1–2 h prior to sample collection.

### Sample collection

At this appointment, the study was again explained to the patient and a signed consent form collected. No further probing was undertaken, to avoid falsifying the test results due to bleeding. The samples collected were in the following order: unstimulated saliva, stimulated saliva, saliva directly from the parotid gland, saliva from the submandibular gland, GCF from the deepest pocket in each quadrant, followed by bacterial sampling with a paper point and GFC from a sulcus (healthy site) in each quadrant. For the healthy control group, similar sampling was undertaken, with the exclusion of pockets.

Unstimulated saliva was collected by placing the patient in an upright position with his head inclined forward so that the produced saliva could be collected by letting the saliva drop into a disposable collection container (Polystyrol PS, 30 ml, Semadeni Plastics Group, Ostermundigen, Switzerland) for a period of up to 15 min, until at least 3 ml of saliva was produced. Following that, the 3 ml of stimulated saliva was also collected after asking the patient to chew for approximately 5 min on a piece of parafilm film (Bemis Company Inc. Oshkosh, WI, USA). To ensure accurate results, the patient was asked to swallow the first portion of saliva before collecting the sample [31–35]. Finally, 2 ml of saliva from each collection container were transferred using fresh disposable pipettes (PE-LD 3.5 ml, Semadeni Plastics Group, Ostermundigen, Switzerland) to individual Eppendorf tubes (Eppendorf AG, Hamburg, Germany) for further analysis. To gain saliva directly from the saliva producing glands, the lips and the cheeks were first isolated from teeth and tongue with cotton rolls. The maxillary teeth were isolated first and the saliva gently removed from the outside of the Stensen’s Duct with the dental unit air syringe. Parotid gland fluid was collected by placing a calibrated volumetric disposable sterile micropipette (minicaps, Hirschmann Laborgeräte GmbH & Co., Eberstadt, Germany) in contact with the Stensen’s duct orifice for 1–2 min until the micropipette was filled (~ 50 µl). Likewise, the saliva from the sublingual and submandibular gland was collected from the Wharton’s duct orifice until the micropipette was filled. Once full, each micropipette was placed in a separate Eppendorf tube containing protease inhibitor solution (Sigma-Aldrich, St Louis, MO, USA).

For the GCF collection, the immediate area from which the sampling was to be done was isolated with cotton rolls and kept dry with the dental unit suction attachment (quadrant-wise). The deepest pocket present in each quadrant had been previously identified and the adjacent tooth was freed of supragingival plaque using a cotton pellet. GCF samples were then obtained using sterile filter paper strips (Periopaper gingival fluid collection strips, Oraflow, Smithtown, NY, USA) inserted into the pocket for 30 s, removed and placed in individual Eppendorf tubes filled with protease inhibitor solution as described above. Care was taken to avoid physical irritation of the sulcular or junctional epithelia. In case the strip of filter paper was contaminated with plaque or saliva, the paper was discarded [31–35]. If the filter paper showed blood, it was likewise discarded and sampling was repeated at the second deepest pocket in that quadrant. As an intrasubject control in the periodontitis group, another four GCF samples were collected, one per quadrant, from healthy sulci of less than 4 mm probing depth without symptoms of gingivitis. Once all samples were collected, they were deep freeze stored at − 80 °C and thawed for analysis within 6 months of collection.

Finally, microbiological sampling was done by placing a sterile paper point (IAI PadoTest, Institut für Angewandte Immunologie IAI AG, Zuchwil, Switzerland) in the deepest pocket per quadrant (if bleeding due to GCF collection occurred, the second deepest pocket was used) for 10 s. All 4 samples were pooled and sent in the packaging provided to the company’s external laboratory for analysis. This RNA-based assay (IAI Pado Test 4.5) tests for *Aggregatibacter actinomycetemcomitans (Aa), Tannerella forsythia (Tf), Porphyromonas gingivalis (Pg), and Treponema denticola (Td), Prevotella intermedia (Pi) and Filifactor alocis (Fa),* which are represented in percentage (%) if at least one site was revealed positive.

### Measurements of the salivary and pockets biomarkers

Samples aliquots from GCF, the major salivary glandular fluids and saliva (stimulated and unstimulated) were thawed on ice and centrifuged at 10,000 rpm for 5–10 min at 4 °C to remove insoluble debris or oral mucosal cells from the supernatants. Commercial enzyme-linked immunosorbent assay (ELISA) kits were used to evaluate levels of aMMP-8 (ab219050) and Lactoferrin (ab200015) following manufacturer’s instructions (ELISA kits, Abcam, Cambridge, UK), each at a dilution of 1:100. The absorbance at 450 nm was accounted for each ELISA on a microplate reader (EZ Read 400 Microplate Reader; Biochrom, Cambourne, UK) and the absorbance reference value (540 or 570 nm) was subtracted from the test values. Experiments were performed on three specimens from each test group in order to confirm the dilution factor of each biomarker. All the experiments were conducted in triplicate.

### Statistical analysis

The data were explored and summarized using descriptive statistics (mean, standard deviation, median and interquartile range) and graphical methods. Due to heteroscedastic data, the differences in aMMP-8 and Lactoferrin concentrations between the sources (stimulated and unstimulated saliva, parotid and submandibular glands, pockets and gingival crevicular fluid) were tested pairwise using nonparametric Wilcoxon signed-rank tests. *P*-values were adjusted for multiple testing according to Holm. Comparisons between the healthy and periodontitis group were statistically assessed using Wilcoxon rank sum tests. Moreover, potential associations between standard clinical parameters, microbial assessments and aMMP-8 and Lactoferrin were investigated. All plots and tests were calculated with the statistical software R (R Core Team, 2018, R Foundation for Statistical Computing, Vienna, Austria).

## Results

### Clinical evaluation and sample collection

The data of individual recruited participants is listed in Table 1. In brief, this study evaluated ten participants, 30–69 years of age, with periodontitis and ten systemically and periodontally healthy participants, 17–59 years of age. A total of 160 samples (n = 8/subject) were collected for analysis. For periodontitis patients, the mean PPD was 7.15 mm, with a range between 5 and 12 mm and the mean sulcus depth (healthy sites) was 2.95 mm. For the healthy patients, the mean collective sulci was 2.5 mm, with a range between 2 and 3 mm and the mean sulcus depth was 2.7 mm. The healthy patients exhibited limited BOP, PI with clinical CAL not exceeding 3 mm. The patients with periodontitis exhibited higher BOP and PI with generalized CAL ranging from 6 to 12 mm. Next, the periodontal inflamed surface area (PISA) and periodontal epithelial surface area (PESA) were recorded on a Microsoft Excel spreadsheet and calculated using the formulas previously defined [36]. PISA ranged from 22.70 mm^2^ (≈ 0.2 cm^2^) in healthy individuals, to 5670 mm^2^ (≈ 56.7 cm^2^) in patients with chronic generalized periodontitis. Further, PESA ranged from 1125 mm^2^ (≈ 11.25 cm^2^) in healthy individuals, to 4407 m^2^ (≈ 44.07 cm^2^) in patients with chronic generalized periodontitis patients.

**Table 1:** Data of individual patients and clinical measurements of PPD, probing pocket depth; BOP, bleeding on probing; PI, plaque index; PESA, periodontal epithelial surface area, PISA, periodontal inflamed surface area

Patient	Cigarettes/day	No. of teeth	PPD (mm)	BOP	PI	PISA/PESA
			Mean	%	%	mm^2^
1	0	25	5.2	42	76	2537.2/1222.3
2	1	23	7.2	44	62	5670.1/3422.6
3	10	22	6.5	31	75	2074.1/678.7
4	0	31	8.9	36	78	2086.5/827.5
5	0	30	6.4	48	69	3523.9/2268.9
6	0	24	5.9	46	55	2268.9/2030.2
7	0	29	8.5	79	68	5516.4/4407.7
8	1	26	7.3	62	84	2818.2/1972.9
9	8	28	5.9	54	75	3797.7/2331.3
10	0	26	6.6	31	74	2461.8/925.4
11	3	24	N/A	20	25	28.6/1125.3
12	0	27	N/A	20	25	25.9/1254.3
13	0	30	N/A	24	20	30.2/1599.2
14	0	32	N/A	15	25	25.3/1892.4
15	0	28	N/A	10	15	22.7/1793.3
16	0	28	N/A	15	25	28.9/1299.3
17	0	24	N/A	22	30	26.9/1923.3
18	0	28	N/A	20	27	31.2/2014.2
19	0	28	N/A	25	25	27.9/1892.2
20	0	30	N/A	21	20	28.1/1643.4

### Measurements of the salivary and pockets biomarkers

Concentration levels (ng/ml) of aMMP-8 and Lactoferrin from the different locations were tested using ELISA (Table 2). Pocket depth values less than 4 mm are considered as healthy periodontium. Accordingly, GCF from sulci (≤ 4 mm) in periodontitis patients were evaluated to identify healthy aMMP-8 concentrations. One previous study reported that a range of 0–7.4 ng/ml of aMMP-8 of eluate was found in healthy controls [31]. Thus, sites with ≥ 8 ng/mL used here are considered to indicate sites with breakdown of collagenolytic tissue, which shows gingivitis and/or periodontitis-affected sites.

**Table 2:** Concentrations of aMMP-8 and lactoferrin in sulci and deep pockets

Patient	aMMp-8 (ng/ml of eluate)			Lactoferrin (ng/ml of eluate)		
	Mean pockets	Mean Sulci	% $\ge$ 8 ^a^	Mean pockets	Mean Sulci	% $\ge$ 8^a^
1	26.03	21.78	78.60	1763.70	1324.10	49.30
2	31.42	21.05	75.20	1748.50	1323.30	52.30
3	37.77	16.82	46.50	1709.90	1270.30	61.90
4	36.67	14.36	29.60	1740.30	1150.40	58.80
5	37.17	15.02	45.30	1694.30	1232.70	42.10
6	34.54	13.89	58.30	1701.60	1279.75	35.90
7	34.64	15.11	44.30	1679.90	1339.25	45.80
8	39.04	13.33	36.50	1728.20	1274.75	65.80
9	36.33	13.57	48.60	1428.70	1191.40	34.60
10	35.35	13.24	58.60	1365.20	1130.05	57.30
11	N/A	5.34	N/A	N/A	508.6	N/A
12	N/A	4.78	N/A	N/A	509.2	N/A
13	N/A	5.24	N/A	N/A	510.5	N/A
14	N/A	5.78	N/A	N/A	507.2	N/A
15	N/A	5.29	N/A	N/A	510.9	N/A
16	N/A	4.91	N/A	N/A	509.3	N/A
17	N/A	4.72	N/A	N/A	508.8	N/A
18	N/A	4.71	N/A	N/A	510.1	N/A
19	N/A	4.97	N/A	N/A	511.9	N/A
20	N/A	5.12	N/A	N/A	505.9	N/A

aMMP-8: active matrix metalloproteinase-8

^a^% ≥ 8 ng/mL aMMP‐8 or Lactoferrin: percentage of collagenolysis‐prone sites according to Prescher et al. [31]

**Fig. 1:**
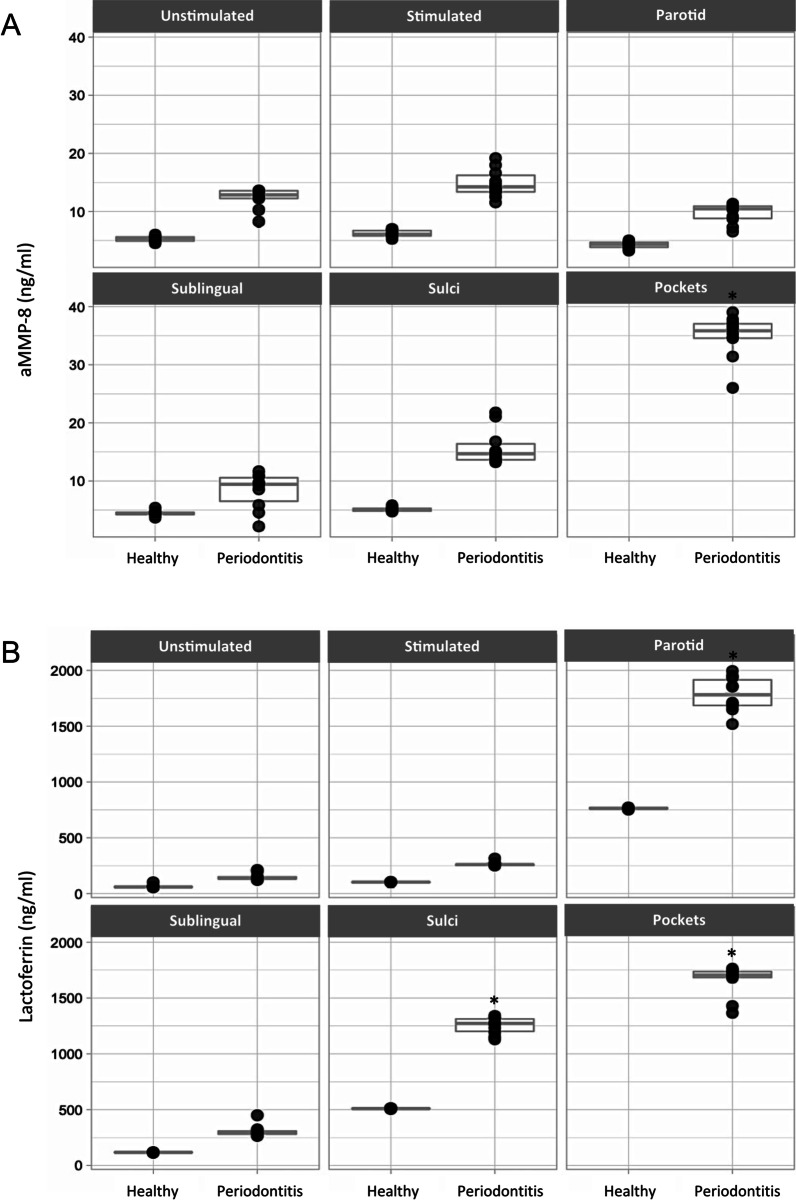
Concentration levels of **a** aMMP-8 (ng/ml) and **b** Lactoferrin (ng/ml), from all sites tested: unstimulated saliva, stimulated saliva, parotid, sublingual, pockets and GCF in both healthy and periodontitis patients. *P < 0.05

The results showed that aMMP-8 was above the detection limit at all tested sites, in the range of 1.5–41.5 ng/ml of eluate across all patients. Also, aMMP-8 levels were found to be significantly higher in the deep pockets in periodontitis patients (mean = 34.9 ng/ml) compared to all other sources (unstimulated saliva, stimulated saliva, parotid, sublingual and GCF from sulci, *P* = 0.03, Fig. 1a). The concentration of aMMP-8 was significantly lower in healthy patients in all of the analyzed sites compared to periodontitis patients (*P* < 0.05 each; Fig. 1a). Lactoferrin was also present at all sites in periodontitis patients, in the range of 121.2–1995.9 ng/ml of eluate. Lactoferrin in periodontitis patients was found to be significantly higher in the parotid (mean = 1785 ng/ml) compared to sublingual glandular fluid (mean = 305.9 ng/ml, *P* = 0.03). Higher values of Lactoferrin were also found in the deep pockets (mean = 1656 ng/ml) compared to GCF from the same patients’ sulci (mean = 1252 ng/ml, Fig. 1b). The concentration of Lactoferrin was significantly lower in healthy patients compared to the periodontitis patients in all of the analyzed sites (*P* < 0.05 each; Fig. 1b).

**Fig. 2:**
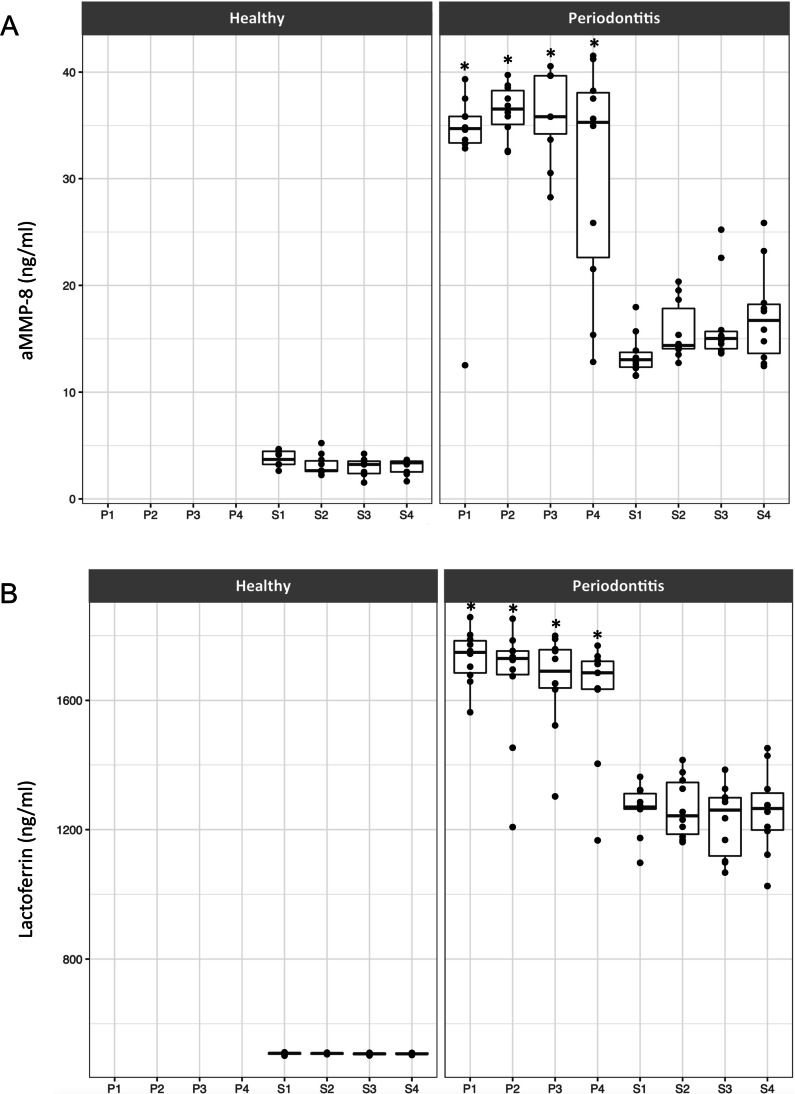
Site-specific correlations of aMMP-8 (**a**) and Lactoferrin (**b**). Boxplots demonstrating the levels of **a** aMMP-8 (ng/ml) and **b** Lactoferrin (ng/ml) total protein for each individual deep pocket (P1–P4) and sulcus (S1-S4) tested in healthy and periodontitis patients. *P < 0.05

Additional information to Fig. 1 is seen in Fig. 2, which presents the individually shown site-specific concentrations of aMMP-8 and Lactoferrin. The mean site-specific concentrations of aMMP-8 and Lactoferrin were found to be significantly higher in deeper pockets (P) 1, 2, 3 and 4 (P1, P2, P3 and P4) compared to sulci (S) 1, 2, 3 and 4 (S1, S2, S3 and S4) in periodontitis patients (*P* = 0.002; Fig. 2a, b). Further, healthy patients presented significantly lower concentrations of aMMP-8 (mean = 3.2 ng/ml) and Lactoferrin (mean = 507.5 ng/ml) in their sulci compared to the sulci of periodontitis patients (aMMP-8: mean = 15.7 ng/ml), Lactoferrin: mean = 1253 ng/ml).

**Fig. 3:**
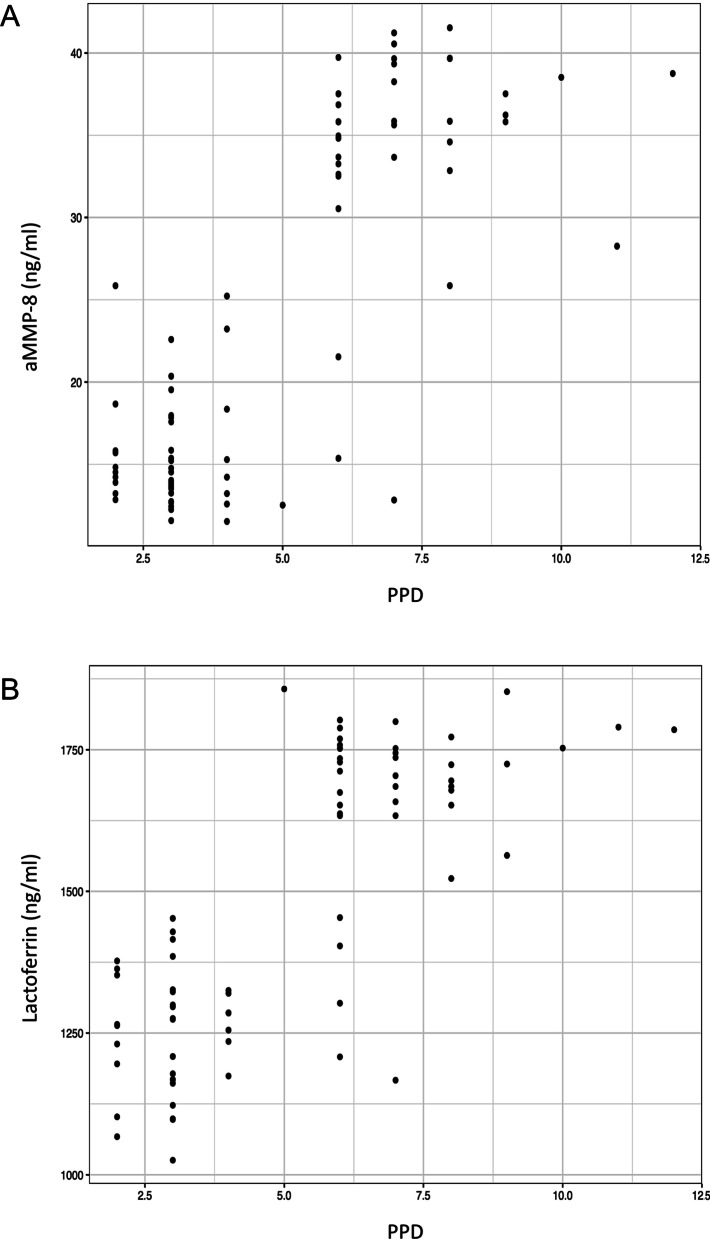
Site-level measurements of MMP-8 (**a**) and Lactoferrin (**b**) of aMMP-8 versus probing pocket depth (PPD)

No association was observed between either aMMP-8 or Lactoferrin and the subjects’ age, sex, smoking status, number of missing teeth, tooth mobility, exudate, CAL or bleeding index. In addition, no relationship was evident between presence of subgingival microorganisms (*Aa, Tf, Pg, Td, Pi, Fa*) and the levels of aMMP-8 or Lactoferrin. However, positive relationships were found between levels of aMMP-8 and PPD, levels of Lactoferrin and PPD and MMP-8 and Lactoferrin (Fig. 3a, b).

## Discussion

Gold standard clinical parameters, i.e. pocket probing and radiographic examination, are widely used in clinical practice for identifying established periodontitis disease, but the early detection efficacy and/or disease prediction using these methods remain inconclusive. Noninvasive detection of salivary biomarkers, such as aMMP-8 and Lactoferrin, may provide a helpful solution to determine early disease manifestation and predict the likelihood of future periodontal breakdown, as they have been found to be increased enzymes in established advanced periodontal disease [18, 19]. Since the biological phenotype of the periodontitis patient is neither properly reflected by the clinical assessment methods nor show host response with subsequent inflammatory burden, an early biomarkers detection may lead to a more successful treatment, which shows its clinical significance for patients [18]. In fact, periodontal patients who get treatment for periodontitis in its earliest stages generally find that the interventions are less invasive and disruptive.

Although several studies have correlated biomarkers with periodontitis and its progression [37, 38], the actual route(s) by which these biomarker enzymes find their way into saliva have not been assessed yet according to the authors’ knowledge. And as source of any oral biomarker should be taken into account when analyzing its function, and MMP-8 and Lactoferrin could possibly be derived from a number of different sources including GCF as well as secretion from salivary glands. Whole saliva represents a complex fluid mixture, and gingival crevice exudate accounts only for a part of its composition, while another important part comes from major salivary glands [14–34]. In addition, the flow rate fluctuates during the day and depends strongly on the stimuli affecting the salivary glands, which may dictate the concentration of its constituents such as MMPs. MMP-8 is considered one of the main collagenases related to connective tissue and alveolar bone destruction and it is considered a crucial mediator of established irreversible periodontal disease [37–39]. Active MMP-8 found in saliva has been used as the biomarker for point-of-care devices for periodontitis, as increases in its level is correlated with severity and status of periodontitis [27, 37–40]. Active MMP-8 in GCF has also been shown to be a potential marker for site-specific diagnosis in order to evaluate the periodontal treatment response [28]. The results of this study demonstrated similar concentrations of aMMP-8 in the parotid and sublingual/submandibular glandular fluids. However, these levels were overall the lowest found and significantly lower than the concentrations in unstimulated saliva, sulcus GCF and stimulated saliva. The highest mean concentrations were found in the periodontal pockets compared to healthy patients. These results indeed highlighted the periodontally diseased pocket as the main source of aMMP-8. One possible explanation of elevated concentrations of aMMP-8 in saliva, well beyond that found in the saliva’s sources (parotid and submandibular glands), is that through the chewing motion associated with the collection of stimulated saliva, sufficient quantities of aMMP-8-containing GCF may be released into the saliva. Ultimately, the saliva can provide a simple painless source for collecting and testing for aMMP-8. Further, these findings corroborate previous studies that identified aMMP-8 in deep pockets and as one of the metalloproteinases that actively participate in the degradation of matrix proteins and destruction of connective tissue and alveolar bone during progression of periodontitis [14, 27, 28, 31]. And also confirms that most collagenases in saliva appears to be originated from polymorphonuclear leukocytes entering the oral cavity through the gingival sulcus [28].

Lactoferrin is a ubiquitous iron-binding protein component of the saliva that is present in secondary granules of polymorphonuclear leukocytes and it is mainly produced in the acute phase of periodontitis [41]. In this study, the levels of Lactoferrin provided a different picture than that of aMMP-8. The highest concentrations, interestingly, were found in the parotid gland samples, followed by deep pockets and gingival sulci. The sublingual glandular fluid, in contrast, displayed significantly lower levels of Lactoferrin. As in previous studies, the concentration of biomarkers in the oral cavity may be related to the glandular fluid composition of saliva [42]. In fact, another study showed that unstimulated saliva is mainly produced in the submandibular gland (70%), followed by parotid (20%) and sublingual glands (2%), while stimulated saliva is mostly produced by the parotid glands (60%) and submandibular gland (30%) [42]. Higher fluid contribution from the parotid to stimulated saliva production, however, does not explain the fact that both stimulated and unstimulated saliva showed the lowest concentrations of Lactoferrin overall. As an iron-binding glycoprotein produced by salivary glands, Lactoferrin has been shown in a previous study to have been released in higher concentrations during an acute phase of gingival inflammation and was readily detected in saliva from periodontal disease patients [43]. Nevertheless, according to the findings of this study, saliva does not appear to be the best source for testing the presence of Lactoferrin as a biomarker for periodontitis. Our findings found higher levels of Lactoferrin in GCF than in saliva. Several studies have also previously identified high levels of Lactoferrin in the GCF of patients with periodontitis and these levels were associated with elevated PPD [41–44]. Even though some fluctuations in the levels of biomarkers were seen in other studies [45], overall, the levels of biomarkers found in the present study were within similar ranges to previously reported [46].

This study has taken a step in determining the main source of major biomarkers related to periodontal disease determination and progression. One may consider a limitation of this study to be the sample size. Practical factors played a large role in the relatively small sample size. Despite recruitment running over a 1-year period, only a limited number of patients were willing to burden themselves with an additional clinical appointment to participate in the study. However, considering the number of sites tested and using a “intrasubject design”, our data provided meaningful results in this limited study population. In fact, the results presented here were quite homogenous. However, future larger scale studies should consider testing GCF for these biomarkers in a cohort that would not have any scheduling hardship when participating (e.g. dental hygiene recall population) over a longer time period (regularly scheduled recall appointments).

## Conclusions

We conclude that aMMP-8 and Lactoferrin, in combination, may be useful as diagnostic and predictive adjunctive biomarkers for periodontitis. High levels of these biomarkers were detected in samples taken from pockets of periodontitis patients. Further, both aMMP-8 and Lactoferrin were elevated in the sites considered being clinically healthy in periodontitis patients. Even though Lactoferrin was present in the GCF (sulci) and parotid gland fluids of healthy subjects, the concentrations were significantly lower than in the GCF of periodontitis patients. Both aMMP8 and Lactoferrin were shown in this study to enter the oral cavity through the gingival sulcus and salivary glands. Further large-scale studies with long-term follow-up to confirm the potential of these biomarkers to predict future, preclinical, inflammatory processes are still needed.

## Data Availability

The datasets used and analyzed in this study are available on reasonable request from the corresponding author.

## Abbreviations

Mmp-8: Metalloproteinases 8
aMMP-8: Activated/active metalloproteinases 8
ELISA: Enzyme-linked immunosorbent assay
GCF: Gingival crevicular fluid
PPD: Probing pocket depths
BOP: Bleeding on probing
CAL: Clinical attachment levels
PISA: Periodontal inflamed surface area
PESA: Periodontal epithelial surface area
P: Deeper pockets
S: Sulci
